# Evaluating the Application of MUSE Diffusion-Weighted Imaging in Esophageal Cancer in Comparison with HR and Single-Shot DWIs

**DOI:** 10.3390/diagnostics16081155

**Published:** 2026-04-13

**Authors:** Ting Dong, Tuo He, Guirong Zhang, Huizhi Mi, Zhanghao Huang, Jianzhong Li, Guangxu Han, Dun Ding

**Affiliations:** 1 Department of Medical Imaging, The Second Affiliated Hospital of Xi’an Jiaotong University, Xi’an 710004, China; dt971823992@126.com (T.D.); guirjyhy@163.com (G.Z.);; 2 Health Science Center, Xi’an Jiaotong University, Xi’an 710004, China; 3 Department of Thoracic Surgery, The Second Affiliated Hospital of Xi’an Jiaotong University, Xi’an 710004, China; 4 MR Research, GE HealthCare, Beijing 100076, China

**Keywords:** diffusion-weighted imaging, magnetic resonance imaging, esophageal cancer, MUSE-DWI

## Abstract

**Background/Objectives:** To evaluate and compare the qualitative and quantitative image performance of multiplexed sensitivity-encoding diffusion-weighted imaging (MUSE-DWI) against conventional single-shot (ss-DWI) and high-resolution single-shot (HR-ssDWI) sequences in patients with esophageal cancer. **Methods:** Twenty patients who underwent esophagus MRI, including ss-DWI, HR-ssDWI and MUSE-DWI, were retrospectively enrolled. Image quality, esophageal contour, lesion conspicuity and image distortion were independently graded by two radiologists using a five-point scale and compared between the three sequences. Signal-to-noise ratio (SNR) and contrast-to-noise ratio (CNR) of esophageal tissue were measured and compared between the three sequences. **Results:** After Bonferroni correction (*p* < 0.017), MUSE-DWI had significantly higher scores than HR-ssDWI in image quality, esophageal contour delineation and lesion conspicuity, and all three sequences had statistically significant differences in image distortion scores with MUSE-DWI performing the best. Quantitative analysis revealed that MUSE-DWI had the highest SNR and CNR values; significant differences were found in SNR between ss-DWI and HR-ssDWI (*p* < 0.001), and in both SNR and CNR between HR-ssDWI and MUSE-DWI (*p* < 0.001), while no significant differences were observed in SNR and CNR between ss-DWI and MUSE-DWI (*p* > 0.017). **Conclusions:** MUSE-DWI outperforms ss-DWI and HR-ssDWI in reducing image distortion, with comparable quantitative image quality metrics to ss-DWI. It represents a valuable optimized DWI technique for esophageal clinical imaging.

## 1. Introduction

Esophageal cancer (EC) ranks as the 11th most common malignancy worldwide and the seventh leading cause of cancer-related mortality, accounting for 2.6% of all new cancer cases and 4.6% of cancer deaths, according to the latest data from the GLOBOCAN 2022 [1]. This highlights the aggressive nature of the disease and the considerable challenges posed by its clinical management. Eca is known for its difficulty in visual detection, necessitating early and precise identification to prevent disease progression and improve patient outcomes [2,3]. Recent studies have also emphasized the clinical importance of Spectrum-Aided Visual Enhancer (SAVE) technology combined with deep learning models in achieving early screening for esophageal cancer [4]. Endoscopy serves as the gold standard for the definitive diagnosis of ECa, whereas magnetic resonance imaging (MRI) offers a valuable non-invasive alternative for lesion assessment and staging [5]. Accumulating clinical evidence has validated the excellent performance of MRI, particularly diffusion-weighted imaging (DWI), in the early evaluation of therapeutic responses to neoadjuvant chemotherapy and concurrent chemoradiotherapy for ECa [5,6,7]. As a non-invasive imaging technique, DWI identifies malignant lesions by detecting abnormal water molecular motion in tumor tissues, and has been widely applied in oncological imaging [8]. In ECa, accurate delineation of tumor boundaries is essential for treatment planning. DWI offers a potential advantage by detecting variations in the tumor’s cellular density and structure [9]. Improved DWI quality is critical for detecting small cancers and accurately delineating lesion extent.

Single-shot echo-planar imaging (EPI) is the conventional technique for clinical DWI acquisition, which captures the entire k-space in a single excitation to minimize motion artifacts via ultra-fast scanning [10]. Nevertheless, single-shot EPI has inherent technical limitations, such as low spatial resolution, motion artifact susceptibility and geometric distortion induced by magnetic field inhomogeneity, which severely hinder the detection and quantitative measurement of small esophageal lesions [10,11]. In addition, it is prone to several limitations, including image distortions due to B0 inhomogeneity at tissue/air interfaces and T2 blurring, both of which place limitations on spatial resolution [12]. Given that the esophagus is adjacent to the trachea, image distortion caused by artifacts in DWI will seriously affect the evaluation of esophageal lesions and hinder the identification of target lesions [13]. High-resolution single-shot DWI (HR-ssDWI) improves spatial resolution by extending EPI readout time, but this strategy requires higher parallel imaging acceleration factors (e.g., SENSE), which in turn exacerbate image artifacts and reduce signal-to-noise ratio (SNR), thus negating the advantages of enhanced spatial resolution [14].

Given the inherent limitations of conventional imaging, an advanced DWI technique capable of providing superior image quality is essential for the precise evaluation and longitudinal monitoring of esophageal lesions. Multiplexed sensitivity-encoding DWI (MUSE-DWI) is an optimized multi-shot EPI technique that enhances spatial resolution and mitigates geometric distortion by segmenting k-space into multiple interleaved segments along the phase-encoding direction [15]. By acquiring image data across multiple k-space segments, MUSE-DWI shortens the echo time (TE) extension associated with increased spatial encoding, thus effectively reducing geometric distortion in DWI images [11,16,17]. Numerous preclinical and clinical studies have confirmed that MUSE-DWI can reduce geometric distortion; improve spatial resolution and elevate SNR in various abdominal and breast organs, including the liver, prostate, intestine, pancreas and breast [11,14,18,19,20]. Nevertheless, the role of MUSE-DWI in assessing ECa remains to be fully investigated.

DWI is an important predictor of early diagnosis and pathological response of esophageal cancer [8,21]. Conventional ss-DWI suffers from geometric distortion that may compromise accurate T staging and radiotherapy target delineation in esophageal cancer. We hypothesize that MUSE-DWI, by reducing image distortion and improving spatial resolution, enables clearer delineation of the tumor extent and fine structural details in esophageal cancer, thereby potentially enhancing the accuracy of tumor staging and the efficiency of treatment planning. Therefore, this study aimed to compare MUSE-DWI with conventional ss-DWI and HR-ssDWI in the esophagus in terms of objective and subjective image quality, esophageal contour, lesion conspicuity, and image distortion.

## 2. Materials and Methods

### 2.1. Study Participants

This study was approved by the Institutional Review Board, which waived the requirement for informed consent as the study involved a retrospective review of medical records and images. Twenty-nine consecutive patients with suspected esophageal cancer were enrolled in this study. All the patients underwent chest MRI examination, including ss-DWI, HR-ssDWI and MUSE-DWI scans, between October 2023 and October 2024. The MUSE-DWI and HR-ssDWI sequences were included in the routine esophageal MRI protocol because their availability at this institution was limited to certain time periods. Of these, 5 patients were excluded because of severe motion artifacts and 4 patients were excluded due to unclear lesions. Therefore, 20 patients (16 males, age range, 43–85 yrs; mean age, 68.0 yrs) were included as study subjects. There were two types of esophageal cancer (including 19 cases of squamous cell carcinoma and 1 case of adenocarcinoma), and the tumor location (1 case in the upper esophagus, 16 cases in the middle esophagus and 3 cases in the lower esophagus).

The inclusion criteria: (1) patients with histopathologically confirmed esophageal cancer (squamous cell carcinoma or adenocarcinoma); (2) tumors clearly visible on at least one DWI sequence to allow ROI placement; (3) complete clinical and pathological records available for review. The exclusion criteria included: (1) patients who received neoadjuvant chemotherapy, radiotherapy, or chemoradiotherapy prior to MRI examination; (2) poor image quality due to severe motion artifacts, susceptibility artifacts, or low signal-to-noise ratio that precluded diagnostic evaluation; (3) tumors not clearly identifiable on any of the three DWI sequences; (4) incomplete clinical data or inability to confirm tumor location/histology; (5) contraindications to MRI (e.g., pacemaker, claustrophobia, metallic implants).

### 2.2. Image Acquisition

All the MRI examinations were performed on a 3.0-T MR scanner (SIGNA Architect, GE Healthcare, Waukesha, WI, USA) with standard thoracic receive coils. The detailed scan parameters of the three DWI sequences (ss-DWI, HR-ssDWI, and MUSE-DWI) are summarized in Table 1, with b-values set to 0 and 800 s/mm^2^ for all the sequences in accordance with our institutional clinical protocol. Motion correction was performed using respiratory triggering.

### 2.3. Qualitative Analysis

The three DWI sequences (ss-DWI, HR-ssDWI, and MUSE-DWI) from each patient were randomly distributed across three datasets for subjective evaluation, along with randomization of patient order. The two radiologists were blinded to the sequence type during image evaluation. All the images were anonymized and presented in random order. However, because the study was designed to compare three specific DWI techniques, the readers were aware that images from these three sequences were included, but they did not know which image belonged to which sequence at the time of scoring. The same reader performed a second blinded scoring session on the same set of images after a 4-week interval, and the intra-class correlation coefficient (ICC) was calculated to assess intra-observer agreement. Two radiologists with 10 years of experience in thoracic imaging independently evaluated DWI sequences (b = 800 s/mm^2^) for image quality, esophageal contour, lesion conspicuity, and image distortion. A 5-point Likert scale was used for scoring: image quality (1 = poor, 2 = below average, 3 = average, 4 = above average, and 5 = excellent); esophageal contour (1 = undetectable, 2 = poorly defined, 3 = moderately defined, 4 = well-defined, and 5 = sharply defined); lesion conspicuity (1 = invisible, 2 = faintly visible, 3 = moderately conspicuous, 4 = conspicuous, and 5 = highly conspicuous); and image distortion (1 = unacceptable, 2 = severe, 3 = moderate, 4 = mild, and 5 = minimal). Both readers were blinded to the type of DWI sequence (ss-DWI, HR-ssDWI, or MUSE-DWI).

### 2.4. Quantitative Analysis

**Figure 1:**
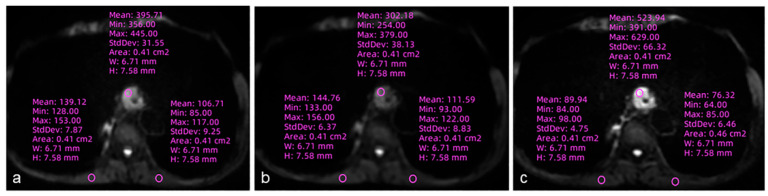
Images acquired from a 56-year-old male esophageal cancer patient with ss-DWI (**a**), HR-ssDWI (**b**), and MUSE-DWI (**c**) sequences. The size and anatomical location of the regions of ROIs in the three DWI sequences remained consistent and exactly the same. The muscle is obtained by taking the average of the signal intensities on both the left and right sides in the same layer.

A quantitative image analysis was performed using software, and ROI linkage was performed by two radiologists using the GE Advantage Workstation (AW, version 4.7, GE Healthcare, Waukesha, WI, USA). Due to geometric distortion in some sequences, the ROI positions were manually calibrated by the radiologists. All the DWI sequences used the same FOV and scanning bed to ensure spatial consistency. ROIs were located in the esophageal carcinoma at the maximum diameter, avoiding necrotic tissue, large vessels, osseous structures and the esophageal cavity. To ensure consistency, the size and anatomical positioning of the ROIs were identical and replicated across all three DWI sequences (Figure 1). The signal intensity of the lesion and the normal esophageal wall (SI and SIn, respectively) and standard deviation (SD) of the muscle background signal were recorded for all the sequences. The SD is calculated as the average of the SD values on the left and right sides of the trapezius muscles. For extensive tumors, ROIs for the normal esophageal wall were placed on anatomical images in esophageal segments clearly distant from the tumor, carefully avoiding any areas of suspected tumor infiltration.

Signal-to-Noise Ratio (SNR): SNR was calculated by dividing the signal intensity of the lesion by the standard deviation of muscle background noise.

SNR=SI/SD

where SI is the average signal intensity of the esophageal lesion area. SD represents the standard deviation of the signal intensity of the trapezius muscle.

Contrast-to-Noise Ratio (CNR): CNR between the lesion and normal tissue was calculated as the difference between the signal intensity of the lesion and the signal intensity of the esophageal wall, divided by the standard deviation of muscle background noise.

CNR=(SI−SIn)/SD

where SI and SIn represent the average signal intensities of the esophageal lesion area and the esophageal normal tissue, respectively. SD is the standard deviation of the signal intensity of the trapezius muscle.

### 2.5. Statistical Analysis

All qualitative and quantitative imaging indices of ss-DWI, HR-ssDWI and MUSE-DWI were compared on a per-patient basis. Since the qualitative scores were non-parametric data, Friedman’s test was used for overall inter-sequence comparison, followed by the Wilcoxon signed-rank test with Bonferroni correction (*n* = 3) for post hoc pairwise comparisons. The quantitative indices (SNR and CNR) were also compared using the same statistical approach (Friedman’s test + Bonferroni-corrected Wilcoxon signed-rank test). Inter-reader agreement for all the qualitative indices was evaluated using Cohen’s kappa coefficient, with the following grading criteria: poor (κ ≤ 0.00), slight (κ = 0.01–0.20), fair (κ = 0.21–0.40), moderate (κ = 0.41–0.60), good (κ = 0.61–0.80) and excellent (κ = 0.81–0.99). Statistical significance was set at *p* < 0.017 after Bonferroni correction for multiple comparisons. The intraclass correlation coefficient (ICC) was used to analyze the intra-observer consistency of two readers for four qualitative indicators and two quantitative indicators. ICC values > 0.75 were considered indicative of good agreement. Statistical analysis was performed using SPSS (version 24).

## 3. Results

### 3.1. Qualitative Analysis

**Figure 2:**
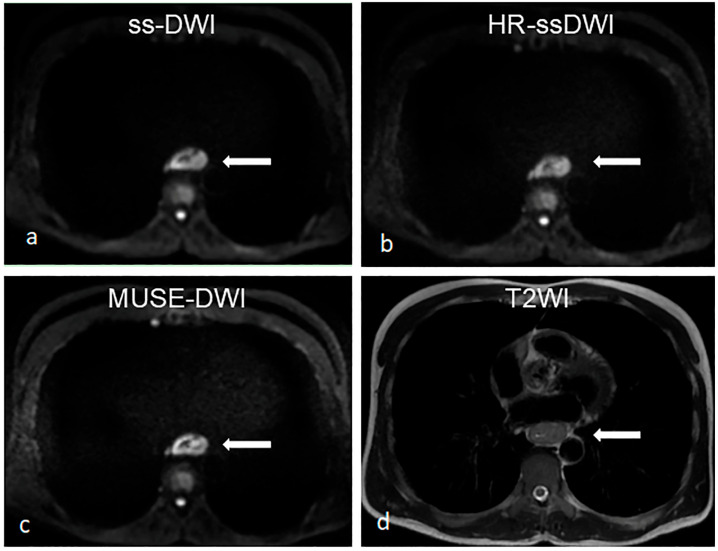
Images acquired from a 48-year-old male esophageal cancer patient with ss-DWI (**a**), HR-ssDWI (**b**), MUSE-DWI (**c**), and T2-weighted (**d**) sequences; the image quality, esophageal contour, lesion conspicuity and image distortion of MUSE-DWI are all superior to HR-ssDWI. Compared with ss-DWI, MUSE-DWI provides a better visualization of fine structures. The arrow indicates the esophageal tumor.

**Figure 3:**
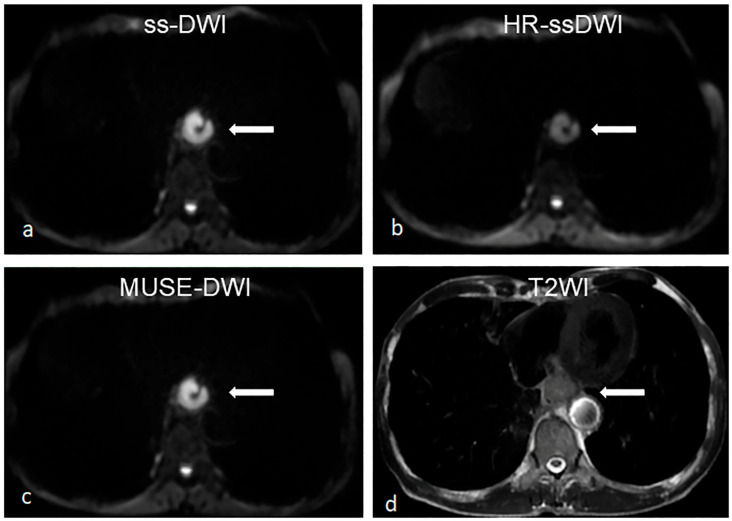
Images acquired from a 56-year-old male esophageal cancer patient with ss-DWI (**a**), HR-ssDWI (**b**), MUSE-DWI (**c**), and T2-weighted (**d**) sequences. There is no significant difference in image quality, esophageal contour and lesion conspicuity between MUSE-DWI and ss-DWI. However, compared to HR-ssDWI, both have better lesion conspicuity and lower image distortion. The arrow indicates the esophageal tumor.

Inter-observer agreement was assessed by the kappa coefficient, with the k values ranging from 0.506 to 0.779, indicating good consistency between the two readers (Table 2). The four qualitative metrics demonstrated excellent intra-observer agreement (all ICCs > 0.75) (Table S1). The results showed that MUSE-DWI yielded a significantly higher score than HR-ssDWI in image quality (*p* = 0.004), while no statistically significant differences were found between ss-DWI and HR-ssDWI (*p* = 0.372), or between ss-DWI and MUSE-DWI (*p* = 0.506) (Table 3). For esophageal contour delineation, MUSE-DWI also presented a markedly superior score compared with HR-ssDWI (*p* = 0.004), with no notable differences between ss-DWI and HR-ssDWI (*p* = 0.319) or ss-DWI and MUSE-DWI (*p* = 0.045). In terms of lesion conspicuity, significant differences were observed between ss-DWI and HR-ssDWI (*p* = 0.012), as well as between HR-ssDWI and MUSE-DWI (*p* = 0.001), whereas no statistical difference was detected between ss-DWI and MUSE-DWI (*p* = 0.149). Regarding image distortion, pairwise comparisons revealed significant differences among all three sequences: ss-DWI vs. HR-ssDWI (*p* = 0.002), ss-DWI vs. MUSE-DWI (*p* = 0.007), and HR-ssDWI vs. MUSE-DWI (*p* < 0.001) (Figure 2 and Figure 3).

### 3.2. Quantitative Analysis

**Figure 4:**
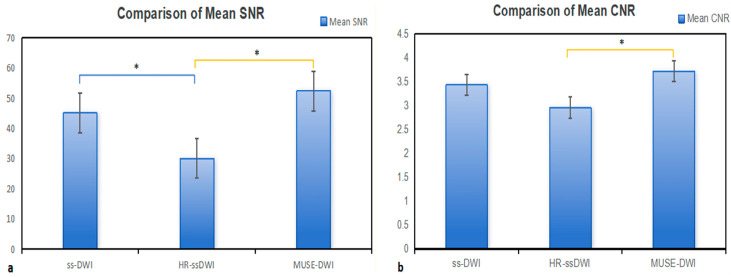
Quantitative SNR and CNR comparisons among three DWI sequences: (**a**) Comparison of mean SNR values among ss-DWI, HR-ssDWI and MUSE-DWI. (**b**) Comparison of mean CNR values among the three sequences. * *p* < 0.017, Bonferroni post hoc test vs. the indicated group.

The two quantitative metrics demonstrated excellent intra-observer agreement (all ICCs > 0.75) (Table S1). Signal-to-noise ratio (SNR) and contrast-to-noise ratio (CNR) were calculated for the three DWI sequences at b = 800 s/mm^2^ to conduct quantitative image assessment, and intergroup comparisons were performed with Bonferroni correction (*p* < 0.017). For SNR, statistically significant differences were found between ss-DWI and HR-ssDWI (*p* < 0.001), and between HR-ssDWI and MUSE-DWI (*p* < 0.001), while no significant difference was noted between ss-DWI and MUSE-DWI (*p* = 0.364) (Table 4 and Table 5). As for CNR, only the comparison between HR-ssDWI and MUSE-DWI showed a significant difference (*p* < 0.001), and no statistical differences were identified between ss-DWI and HR-ssDWI (*p* = 0.037), or between ss-DWI and MUSE-DWI (*p* = 0.153). The mean SNR of HR-ssDWI (30.08 ± 13.37) was significantly lower than that of ss-DWI (45.11 ± 22.51) and MUSE-DWI (52.38 ± 34.01). MUSE-DWI demonstrated the highest mean CNR (3.72 ± 1.33), followed by ss-DWI (3.43 ± 1.41) and HR-ssDWI (2.95 ± 0.96) (Figure 4).

## 4. Discussion

Diffusion-weighted imaging (DWI) is an indispensable sequence in esophageal magnetic resonance imaging (MRI), and acquiring high-quality DWI is critical for the accurate diagnosis and therapeutic response evaluation of esophageal cancer (ECa) [8,22]. The results of this study confirm that MUSE reconstruction technology can significantly improve the quality of esophageal DWI images by reducing image noise, minimizing geometric distortion and enhancing spatial resolution. Notably, qualitative analysis by two experienced radiologists consistently showed that MUSE-DWI outperformed HR-ssDWI in all key imaging indices, including image quality, esophageal contour, lesion conspicuity and image distortion. The inferior imaging performance of HR-ssDWI is attributed to the technical limitations of single-shot EPI: extending the signal readout time to improve spatial resolution enhances T2* modulation of k-space data, which leads to point spread function blurring and reduced bandwidth along the phase-encoding direction, thus exacerbating geometric distortion in esophageal DWI [23]. The readout time for each excitation of the multi-lens EPI is relatively short, which reduces image distortion and blurring. In its shortened echo sequence, MUSE-DWI accumulates smaller phase errors, thereby reducing sensitive artifacts—this is consistent with the previous DWI research results based on multi-lens EPI [14,19,20].

In addition, MUSE-DWI and ss-DWI showed no statistically significant differences in image quality, esophageal contour and lesion conspicuity, which is consistent with previous studies [14]. This finding benefits hospitals unable to perform MUSE-DWI scans, but its reproducibility and stability require further validation in future studies. Importantly, the MUSE-DWI technique provides superior spatial resolution while preserving SNR and CNR equivalent to those of ss-DWI—a distinct technical advantage that merits emphasis. By segmenting k-space into interleaved EPI segments and shortening per-segment echo train length, MUSE minimizes T2 decay and magnetic susceptibility artifacts, enabling high spatial resolutions and better image quality. Without relying on navigator echoes or requiring any pulse sequence modification, MUSE’s reconstruction algorithm is specifically designed to handle and correct for small-scale motion, affording superior SNR [15]. Notably, MUSE-DWI reduced image distortion compared with ss-DWI, suggesting that it represents a promising optimized DWI sequence for esophageal imaging that provides clearer anatomical visualization. Better visualization of the tumor–esophageal wall interface may contribute to more accurate T staging and radiotherapy target delineation. Further large-scale prospective studies are warranted to validate its clinical diagnostic value in esophageal cancer staging and treatment planning. One possible explanation is that MUSE’s multi-shot acquisition reduces geometric distortion and blurring artifacts that are more pronounced in ss-DWI. Signal-to-noise ratio (SNR) is a key quantitative index for evaluating DWI image quality and clinical interpretability. Our quantitative analysis showed that MUSE-DWI had significantly higher SNR and CNR for esophageal cancer lesions than HR-ssDWI, while no statistically significant differences were observed between MUSE-DWI and ss-DWI. In essence, MUSE is an optimized perceptual reconstruction technique that extends the image reconstruction task to the inverse operation of a large matrix (several times the size of the original k-space matrix) [15,24]. This unique reconstruction mechanism enables MUSE technology to effectively counteract the SNR penalty induced by parallel imaging acceleration factors, thus delivering superior image quality and less geometric distortion than HR-ssDWI under the same coil parameters [25]. Therefore, MUSE-DWI not only significantly enhances spatial resolution but also effectively reduces residual aliasing artifacts caused by insufficient reconstruction in parallel imaging, fully demonstrating its technical advantages in esophageal MRI.

This study has several limitations. First, the small sample size and potential selection bias constitute key limitations that may affect the generalizability of our findings. The limited sample size precludes definitive conclusions regarding diagnostic accuracy. Larger prospective multicenter studies are warranted to evaluate the clinical diagnostic value of MUSE-DWI in esophageal cancer. Second, the limited number and subtype diversity of ECa cases may restrict the generalizability of our results, which require validation in multi-center studies encompassing broader patient populations. Third, while DWI is widely used for ECa staging, we did not assess diagnostic performance (e.g., sensitivity, specificity, or staging accuracy) in esophageal cancer and the clinical diagnostic value of the technical advantages of MUSE-DWI needs to be verified by subsequent large-sample prospective studies. Fourth, the acquisition time of MUSE-DWI was prolonged compared with conventional ss-DWI owing to its multi-shot acquisition strategy—an inherent trade-off between image quality and scan efficiency. Future optimization of acquisition parameters is warranted to facilitate clinical adoption. Finally, the use of a single-vendor scanner may limit the generalizability of our findings; thus, validation across multiple imaging platforms is necessary to establish the universal applicability of MUSE-DWI.

## 5. Conclusions

MUSE-DWI outperforms ss-DWI and HR-ssDWI in reducing image distortion, with comparable quantitative image quality metrics to ss-DWI. It represents a valuable optimized DWI technique for esophageal clinical imaging.

## Figures and Tables

**Table 1:** Acquisition parameters of the three DWI techniques.

	ss-DWI	HR-ssDWI	MUSE-DWI
b value (s/mm^2^)	0, 800	0, 800	0, 800
Echo time (ms)	Minimum	Minimum	Minimum
Breathing schemes	RT	RT	RT
Phase-encoding direction	A-P	A-P	A-P
Number of echoes	1	1	1
Number of shots	1	1	2
Slice thickness (mm)	4	4	4
Matrix	128 × 128	192 × 192	192 × 192
FOV (mm)	300 × 300	300 × 300	300 × 300
Bandwidth (kHz)	250	250	250
Pixel Size	2.3 × 2.3	1.6 × 1.6	1.6 × 1.6
Multi NEX	1, 6	1, 6	1, 6
Parallel imaging	ASSET (phase factor 2)	ASSET (phase factor 2)	ASSET (phase factor 2)
Approximate scan time (min: s)	3 min 32 s	5 min 09 s	5 min 34 s

RT: respiratory triggering. Due to respiratory triggering (RT), the actual acquisition time varied among patients depending on individual breathing patterns. The reported values represent the approximate scan duration.

**Table 2:** Qualitative evaluation of the three DWI sequences for image quality, esophageal contour, lesion conspicuity, and image distortion.

	ss-DWI (b800)		κ	HR-ssDWI (b800)		κ	MUSE (b800)	κ	
	Reader 1	Reader 2		Reader 1	Reader 2		Reader 1	Reader 2	
Image quality	4 (5-3)	4 (5-3)	0.637	4 (4-3)	4 (4-3)	0.691	4 (5-3)	4 (5-4)	0.677
	3.88 ± 0.91	3.90 ± 0.87		3.76 ± 0.78	3.72 ± 0.79		3.98 ± 0.94	4.02 ± 0.72	
Esophageal contour	4 (5-3)	4 (5-3)	0.72	4 (5-3)	4 (4-3)	0.70	4(5-3)	4 (5-4)	0.713
	3.80 ± 0.99	3.95 ± 0.99		3.92 ± 0.86	3.8 ± 0.76		4.02 ± 1.01	4.17 ± 0.86	
Lesion conspicuity	4 (5-3)	4 (5-3)	0.779	4 (4.5-3)	4 (4-3)	0.705	4 (5-3)	5 (5-4)	0.775
	4.00 ± 0.91	4.1 ± 0.78		3.84 ± 0.90	3.72 ± 0.79		4.12 ± 1.03	4.27 ± 0.87	
Image distortion	4 (5-3)	4 (5-3)	0.618	4 (5-2)	4 (5-2)	0.585	4 (5-3)	4 (5-3)	0.506
	3.86 ± 0.71	3.73 ± 0.63		3.18 ± 0.80	3.23 ± 0.69		4.32 ± 0.65	4.32 ± 0.57	

Values are expressed as median (IQR); mean + standard deviation.

**Table 3:** Comparison of qualitative image quality, esophageal contour, lesion conspicuity, and image distortion among three DWI sequences.

	ss-DWI vs. HR-ssDWI	ss-DWI vs. MUSE-DWI	HR-ssDWI vs. MUSE-DWI
Image quality	0.372	0.506	0.004
Esophageal contour	0.319	0.045	0.004
Lesion conspicuity	0.012	0.149	0.001
Image distortion	0.002	0.007	0.000

**Table 4:** Quantitative analysis regarding CNR and SNR for the three different DWI sequences.

	ss-DWI (b800)	HR-ssDWI (b800)	MUSE-DWI (b800)
SNR	40.435 (25.673)	27.872 (0.000)	42.885 (31.083)
	45.11 ± 22.51	30.08 ± 13.37	52.38 ± 34.01
CNR	3.489 (0.830)	3.065 (0.014)	3.693 (1.222)
	3.43 ± 1.41	2.95 ± 0.96	3.72 ± 1.33

**Table 5:** *p*-values of qualitatively assessed CNR and SNR comparing three different DWI sequences.

	ss-DWIvs. HR-ssDWI	ss-DWI vs. MUSE-DWI	HR-ssDWI vs. MUSE-DWI
SNR	0.000	0.364	0.000
CNR	0.037	0.153	0.000

## Data Availability

The data presented in this study are available upon request from the corresponding author.

## Supplementary Materials

The following supporting information can be downloaded at https://www.mdpi.com/article/10.3390/diagnostics16081155/s1. Table S1: Intraobserver reliability of qualitative and quantitative image assessment indicators.

## Abbreviations

The following abbreviations are used in this manuscript:

MUSE-DWI	Multiplexed sensitivity-encoding diffusion-weighted imaging
ss-DWI	Single-shot diffusion-weighted imaging
HR-ssDWI	High-resolution single-shot diffusion-weighted imaging
SNR	Signal-to-noise ratio
CNR	Contrast-to-noise ratio
ECa	Esophageal cancer
EPI	Echo-planar imaging
MRI	Magnetic resonance imaging
ROI	Region of interest
SPSS	Statistical Package for the Social Sciences
SD	Standard deviation
