# Supplementary material for: Evaluating the Application of MUSE Diffusion-Weighted Imaging in Esophageal Cancer in Comparison with HR and Single-Shot DWIs
Source: Diagnostics (Basel). 2026 Apr 13;16(8):1155. doi: 10.3390/diagnostics16081155 (PMC13114448; doi:10.3390/diagnostics16081155)
Supplement: Supplementary file 1 [file diagnostics-16-01155-s001.zip › diagnostics-4130419-supplementary.pdf]

Table S1. Intraobserver reliability of qualitative and quantitative image assessment indicators.

**Assessment Indicator**	**Reader1 ICC (95%CI, P-value)**	**Reader2 ICC (95%CI, P-value)**
Image quality	[0.853(0.717～0.927) *P*<0.001]	[0.783(0.595～0.890) P<0.001]
Esophageal contour	[0.767(0.493～0.891) P<0.001]	[0.778(0.583～0.888) P<0.001]
Lesion conspicuity	[0.768(0.566～0.883) P<0.001]	[0.886(0.739～0.934) P<0.001]
Image distortion	[0.793(0.610～0.896) P<0.001]	[0.831(0.674～0.916) P<0.001]
Signal-to-Noise Ratio (SNR)	[0.762(0.559～0.879) P<0.001]	[0.759(0.537～0.880) P<0.001]
Contrast-to-Noise Ratio (CNR)	[0.769(0.568～0.883) P<0.001]	[0.780(0.537～0.896) P<0.001]
